# Assessing reproductive performance and predictive models for litter size in Landrace sows under tropical conditions

**DOI:** 10.5713/ab.23.0406

**Published:** 2024-04-25

**Authors:** Praew Thiengpimol, Skorn Koonawootrittriron, Thanathip Suwanasopee

**Affiliations:** 1 Department of Agricultural Technology, Faculty of Science and Technology, Thammasat University, Pathum Thani 12120, Thailand; 2 Department of Animal Science, Faculty of Agriculture, Kasetsart University, Bangkok 10900, Thailand

**Keywords:** Litter Size, Parity Curve, Piglet Loss at Birth, Prediction Models, Sow Reproductive Performance

## Abstract

**Objective:**

Litter size and piglet loss at birth significantly impact piglet production and are closely associated with sow parity. Understanding how these traits vary across different parities is crucial for effective herd management. This study investigates the patterns of the number of born alive piglets (NBA), number of piglet losses (NPL), and the proportion of piglet losses (PPL) at birth in Landrace sows under tropical conditions. Additionally, it aims to identify the most suitable model for describing these patterns.

**Methods:**

A dataset comprising 2,322 consecutive reproductive records from 258 Landrace sows, spanning parities from 1 to 9, was analyzed. Modeling approaches including 2nd and 3rd degree polynomial models, the Wood gamma function, and a longitudinal model were applied at the individual level to predict NBA, NPL, and PPL. The choice of the best-fitting model was determined based on the lowest mean and standard deviation of the difference between predicted and actual values, Akaike information criterion (AIC), and Bayesian information criterion (BIC).

**Results:**

Sow parity significantly influenced NBA, NPL, and PPL (p<0.0001). NBA increased until the 4th parity and then declined. In contrast, NPL and PPL decreased until the 2nd parity and then steadily increased until the 8th parity. The 2nd and 3rd degree polynomials, and longitudinal models showed no significant differences in predicting NBA, NPL, and PPL (p>0.05). The 3rd degree polynomial model had the lowest prediction standard deviation and yielded the smallest AIC and BIC.

**Conclusion:**

The 3rd degree polynomial model offers the most suitable description of NBA, NPL, and PPL patterns. It holds promise for applications in genetic evaluations to enhance litter size and reduce piglet loss at birth in sows. These findings highlight the importance of accounting for sow parity effects in swine breeding programs, particularly in tropical conditions, to optimize piglet production and sow performance.

## INTRODUCTION

Commercial piglet production relies heavily on the reproductive performance of sows within herds. Farm profitability is often tied to maintaining production levels for cost-efficiency or aligning them with the owner's objectives. To achieve these goals, pig producers must gain insights into their sow's potential and reproductive patterns across different parities. This knowledge empowers them to make informed decisions on culling and replacement, ultimately optimizing their breeding herds' management. While traditional indicators like litter size have been used to gauge sow reproduction, piglet loss at birth can serve as a compelling indicator of both sow welfare and productivity [1].

Sow reproductive performance can exhibit significant variation depending on parity [2,3]. Several studies have underscored the impact of parity on various reproductive indicators, including total born and born-alive piglets, mummified and stillborn piglets, and others [3–6]. For example, 1st parity sows often yield smaller litter sizes, which increase in subsequent parities [7,8]. This inferior 1st parity performance may stem from the sow's reproductive tract's immaturity and incomplete development [9]. Conversely, higher parity sows may experience declining litter size, accompanied by an increase in born-dead piglets, possibly due to poor uterine muscle contraction during delivery, a common occurrence in older sows with multiple parturitions [10].

Litter size and piglet loss at birth patterns across parities are recognized as non-linear, influenced by physiological factors. Low repeatability has been observed for key indicators such as total born piglets, born-alive piglets [11–13], and piglet loss at birth [11,14], implying that using initial performance as a selection criterion may lack accuracy. Thus, finding a suitable mathematical model to describe these patterns is promising for predicting sow piglet productivity. Models like the Legendre polynomial, particularly the 3rd degree polynomial, have proven effective in predicting total born piglets [9]. The longitudinal model, which combines a straight line with a Gaussian curve [15], is another approach that mirrors the lactation curve observed in dairy cattle. It has been suggested as a suitable model for fitting the curve of litter size at different parities. Additionally, the incomplete gamma function, commonly used to describe the lactation curve in dairy cattle, could offer an alternative for modeling litter size patterns across parities in sow populations.

Despite these model options, research on mathematical models describing litter size and piglet loss patterns across parities, particularly in sows raised in tropical conditions, remains limited. Therefore, this study pursues two primary objectives: first, to comprehensively investigate litter size and piglet loss patterns across parities in individual sows; and second, to rigorously identify the optimal mathematical model that best elucidates these patterns in a Landrace sow population—a representative case study in a tropical environment. This research contributes to our understanding of sow reproductive performance in specific environmental contexts and offers valuable insights for improving sow management strategies in similar settings.

## MATERIALS AND METHODS

### Population and data

The study dataset comprising sow identification and reproductive records, totaling 2,322 observations, was gathered from 258 Landrace sows reared on a commercial pig farm located in Northern Thailand (18°28′42.3″ N; 98°47′50.3″ E; elevation 310 m above sea level). To ensure the data's quality and reliability, this study selected only sows that consecutively performed parities 1 to 9 for data analysis, which underwent farrowing between March 2010 and January 2019. Observations from the 10th parity onwards were excluded from analysis due to the limited sample size.

Additionally, replacement gilts displaying successful mating at an age exceeding 15 months were indicative of potential reproductive issues. Consequently, sows initiating their first litter at an age exceeding 550 days were omitted from the dataset to eliminate potential confounding effects linked to underperforming sows.

The reproductive records encompassed vital traits, including the number of total born piglets (NTB), number of born alive piglets (NBA), number of mummified piglets (NMM), and number of stillborn piglets (NSB). Specifically, piglets that did not survive beyond birth were classified as stillborn piglets. The cumulative count of mummified and stillborn piglets was employed as the number of piglet losses at birth (NPL). Furthermore, the proportion of piglet losses at birth (PPL) was calculated using the formula: (NPL/NTB)×100. Notably, NBA was adopted as a surrogate indicator for litter size, while NPL and PPL were employed to elucidate patterns of piglet loss at birth within the Landrace population under investigation.

### Ethics approval

The sows included in this study were integral members of the production herd located on a commercial pig farm that rigorously adhered to the established good agricultural practices outlined by the National Bureau of Agricultural Commodity and Food Standards. The performance data pertaining to these sows were obtained from the farm's systematically collected and meticulously maintained database. Consequently, ethical clearance for the utilization of these sow records in this research was deemed unnecessary, as the study exclusively involved the analysis of pre-existing and non-invasive observational data.

### Feeding and management

Up to October 2015, the sows were accommodated within an open-house system, after which they were transitioned to an evaporative cooling system. In both housing systems, a consistent regimen of feeding, management protocols, and healthcare practices was meticulously followed.

Gilts and non-lactating sows adhered to a daily feeding schedule comprising two feedings at 07:00 and 13:00. Their daily ration consisted of 2.5 kg/d, characterized by 16% crude protein content and metabolizable energy (ME) levels ranging from 3,200 to 3,500 kcal/kg. Conversely, lactating sows received four daily feedings at 07:00, 10:00, 13:00, and 15:00, with a ration ranging from 5 to 6 kg/d. This specialized ration contained 17% to 18% crude protein and an ME content of 4,060 kcal/kg. These standardized feeding practices were meticulously maintained throughout the study period to ensure uniformity in sow management, thereby bolstering the reliability and validity of the research outcomes.

Observations of estrus signs were conducted diligently in replacement gilts and sows, twice daily during morning and afternoon sessions. This comprehensive assessment involved visual appraisal and boar exposure. Replacement gilts were transitioned to mating stalls individually five days before their third estrus cycle. The initial mating for replacement gilts occurred upon reaching 8 to 9 months of age or achieving a body weight of 140 kg. Artificial insemination was meticulously conducted twice within a 12-hour interval following estrus detection by farm personnel, employing semen from the same boar for both insemination events.

After mating, gilts and sows were housed in individual pens, with relocation to farrowing pens occurring approximately one week before the anticipated parturition date. The lactation period was extended for approximately 28 days. Upon weaning, piglets were transitioned to the nursery unit, while sows were relocated to individual pens, preparing them for subsequent production cycles. Comprehensive feeding and management practices were enacted to ensure the optimal health and performance of the sows throughout the research duration.

### Statistical analysis

The reproductive performances of sows, including NBA, NPL, and PPL across parities 1 to 9, were subjected to detailed statistical analysis. The PROC UNIVARIATE function within SAS 9.3 System Options [16] was utilized to statistically describe the data. This included assessing normality of distribution and calculating key descriptive statistics such as mean, standard deviation (SD), and range.

To address the skewed distributions of NPL and PPL, natural logarithm transformations [ln(trait+1)] were applied to enhance normality before testing the significance of the parity effect. These transformed traits were denoted as tNPL and tPPL.

The study period encompassed various seasons categorized as winter (November to February), summer (March to June), and rainy (July to October). These were combined with the year of farrowing to create contemporary groups, resulting in a total of 27 contemporary groups. Sows that farrowed within the same year-season subclass (contemporary group) were assumed to have experienced similar feed regimens, routine management, sanitary programs, and environmental conditions, ensuring consistent grouping for analysis.

To test for significant differences in NBA, tNPL, and tPPL among parities, a mixed model was employed using SAS 9.3 System Options [16]. In this model, farrowing year-season (contemporary group), parity, and age at first farrowing (covariate) were considered fixed effects, while animal and residual factors were treated as random effects. The detailed structure of the mixed model is presented in equation (1):


(1) $$y_{\mathrm{ijkl}}=\mu +{\mathrm{FYS}}_{i}+{\mathrm{PAR}}_{j}+b(\mathrm{AFF})+{\mathrm{Anim}}_{k}+e_{ijkl}$$

where, ***y****_ijkl_* represents the observations for NBA, tNPL, and tPPL, ***μ*** denotes the population mean, ***FYS****_i_* corresponds to the ***i***th farrowing year-season (***i*** = 1 to 27), ***PAR****_j_* pertains to the ***j***th parity of sow (***j*** = 1 to 9), AFF signifies age at first farrowing (in days), b represents the linear regression coefficient of AFF on NBA, tNPL, and tPPL, ***Anim****_k_* represents the random animal effect (***Anim*** ~ ***NID***[0, ***V****_anim_*]), and ***e****_ijkl_* accounts for the random residual (***e*** ~ ***NID***[0, ***V****_e_*]).

The least square means (LSMs) for NBA, tNPL, and tPPL within each fixed-effect subclass were estimated and subjected to comparison using t-tests adjusted with a Bonferroni correction at a significance level of α = 0.05.

For the analysis of actual values of NBA, NPL, and PPL from individual sows across parities 1 to 9, four models were considered: the 2nd degree polynomial, the 3rd degree polynomial, Wood gamma function [17], and the longitudinal model proposed by Toft and Jørgensen [15], represented in equations (2) to (5), respectively.

Model 1: 2nd degree polynomial:


(2) $$y_{t}=b_{0}+b_{1}t+b_{2}t^{2}+e_{t}$$

Model 2: 3rd degree polynomial:


(3) $$y_{t}=b_{0}+b_{1}t+b_{2}t^{2}+b_{3}t^{3}+e_{t}$$

Model 3: Wood gamma function [17]:


(4) $$y_{t}={\mathrm{at}}^{b}\;\exp^{-\mathrm{ct}}$$

Model 4: Longitudinal model [15]:


(5) $$y_{t}=-b_{1}\exp\;[-(t^{2}-1)b_{2}]+b_{3}-b_{4}(t)+e_{t}$$

where, ***y****_t_* signifies the reproductive performance (NBA, NPL, and PPL) at the ***t***th parity, ***t*** denotes the parity of the sow, ***b****_0_* is the intercept, ***b****_1_*, ***b****_2_*, ***b****_3_*, and ***b****_4_* are regression coefficients, ***e****_t_* represents the residual, ***a*** signifies the intercept in Model 3, ***b*** relates to the parameter associated with the increasing rate of the trait until peak time, ***c*** is the parameter linked to the decreasing rate of the trait up to the end of lifetime production (9th parity), and ***exp*** denotes the base of the natural logarithm.

For the Wood gamma function analysis, all trait observations were transformed by taking the natural logarithm [ln(trait+1)]. The parameters (***a***, ***b***, and ***c***) were estimated by fitting the Wood gamma function in log-linear form: ***ln***(***y****_t_*) = ***ln(a)+b ln(t)−ct+e****_t_*; where ***e****_t_* represents the residual. Subsequently, the predicted NBA, NPL, and PPL at the 1st to 9th parities for individual sows were computed using an exponential function, ***y****_t_* = **(*exp*[*ln y****_t_***])−*1***.

The estimations of parameters for the 2nd degree polynomial, the 3rd degree polynomial, and the Wood gamma function were conducted using PROC REG, while the longitudinal model was performed using PROC NLMIXED in SAS [16]. Parameter estimates obtained from these four models were utilized to predict NBA, NPL, and PPL for sows across the 1st to 9th parities. Means for predicted NBA, NPL, and PPL at each parity were calculated and illustrated to demonstrate patterns of litter size and piglet loss at birth among Landrace sows in the population, as compared to the mean for actual observations. Differences between predicted and actual values of NBA, NPL, and PPL, calculated as predicted value minus actual value, were computed for each parity to represent prediction errors. Descriptive statistics including mean, SD, and range of these differences for all traits from each model were computed both overall and by parity. To determine the model with the lowest mean and SD for the differences between predicted and actual values, a general linear model was employed as described in equation (6):


(6) $$y_{\mathrm{ijkl}}=\mu +{\mathrm{FYS}}_{i}+{\mathrm{PAR}}_{j}+{\mathrm{MIODEL}}_{k}+b(\mathrm{AFF})+e_{\mathrm{ijkl}}$$

where, ***y****_ijkl_* represents the difference between predicted and actual values of NBA, NPL, and PPL, ***μ*** denotes the population mean, ***FYS****_i_* signifies the ***i***th farrowing year-season (***i*** = 1 to 27), ***PAR****_j_* pertains to the ***j***th parity of sow (***j*** = 1 to 9), ***MODEL****_k_* corresponds to the model used for prediction (***k*** = 1 to 4), ***AFF*** represents age at first farrowing (in days), ***b*** signifies the linear regression coefficient of ***AFF*** on the difference between predicted and actual values of NBA, NPL, and PPL, and ***e****_ijkl_* accounts for the random residual (***e*** ~ ***NID***[0, ***V****_e_*]). The model with the lowest mean and SD for the difference between predicted and actual values signified high accuracy for predicting the trait across parities.

The model that achieved the lowest mean for the difference between predicted and actual values in predicting NBA, NPL, and PPL across parities at individual level was chosen to assess goodness-of-fit at population level. This assessment was conducted using the Akaike information criterion (AIC) [18] and Schwarz Bayesian information criterion (BIC) [19], calculated as follows: ***AIC*** = ***−2logL+2k***, where ***logL*** is the natural logarithm of the likelihood function and ***k*** is the number of parameters; ***BIC*** = ***−2logL*+*klog*(*n*)**, where ***n*** is the number of observations. The model with the smallest AIC and BIC values was selected as the best-fitting model for describing the pattern of the trait across parities.

## RESULTS

### Patterns of litter size and piglet loss at birth

**Figure 1:**
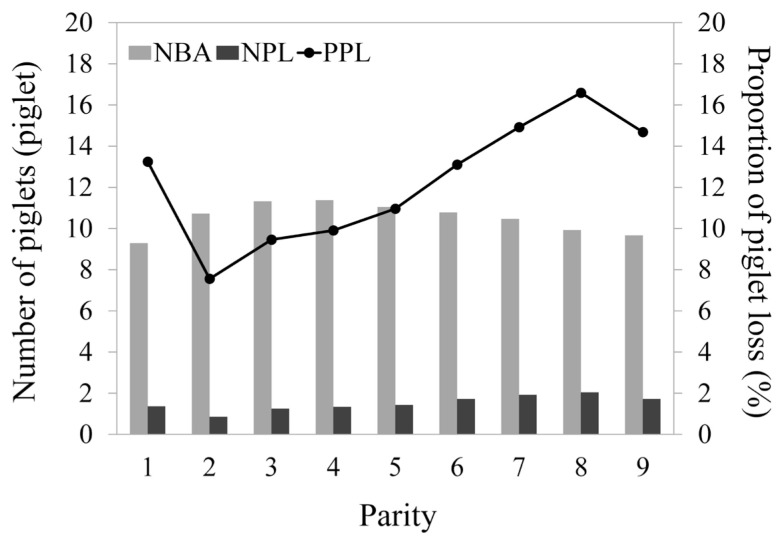
Means for number of born alive piglets (NBA), number of piglet losses at birth (NPL), and proportion of piglet losses at birth (PPL) of sows across parities.

Figure 1 illustrates the dynamic pattern of NBA from the 1st to the 9th parities. Statistical analysis revealed that NBA was significantly influenced by both sow parity (p<0.0001) and the year-season at farrowing (p<0.0001). The 1st parity exhibited the lowest NBA, with an average of 9.29 piglets and a notable degree of variation (SD 3.49 piglets). Subsequently, NBA exhibited a significant increase as parity progressed, reaching its zenith at the 4th parity with an NBA of 11.38 piglets (Table 1). Thereafter, NBA displayed a gradual decline with increasing parity, although it remained relatively high until the 6th parity (10.78 piglets), with no significant differences observed between the 2nd and 4th parities (p>0.05). Starting from the 7th parity, NBA dipped below the overall mean (10.51 piglets) and exhibited a significant decrease from 10.47 piglets in the 7th parity to 9.67 piglets in the 9th parity (p<0.05).

In contrast, the pattern of piglet loss at birth (NPL and PPL) revealed an inverse trend compared to NBA across parities. NPL and PPL exhibited similar patterns from the 1st to the 9th parities in Landrace sows (Figure 1). Both NPL and PPL were significantly influenced by sow parity (p< 0.0001) and the year-season at farrowing (p<0.0001). Mean values for both NPL and PPL were high at the 1st parity (1.37 piglets for NPL and 13.25% for PPL), decreasing to their lowest at the 2nd parity (0.86 piglets for NPL and 7.56% for PPL). Starting from the 3rd parity, NPL and PPL exhibited an increasing trend with the progression of parity, reaching their pinnacle at the 8th parity (with an increase of 0.80 piglets for NPL and a rise of 7.13% for PPL). These values were significantly higher than those observed in sows at the 2nd to 4th parities (p<0.05). However, both NPL and PPL exhibited a slight decrease at the 9th parity.

### Model for describing litter size and piglet loss at birth

**Figure 2:**
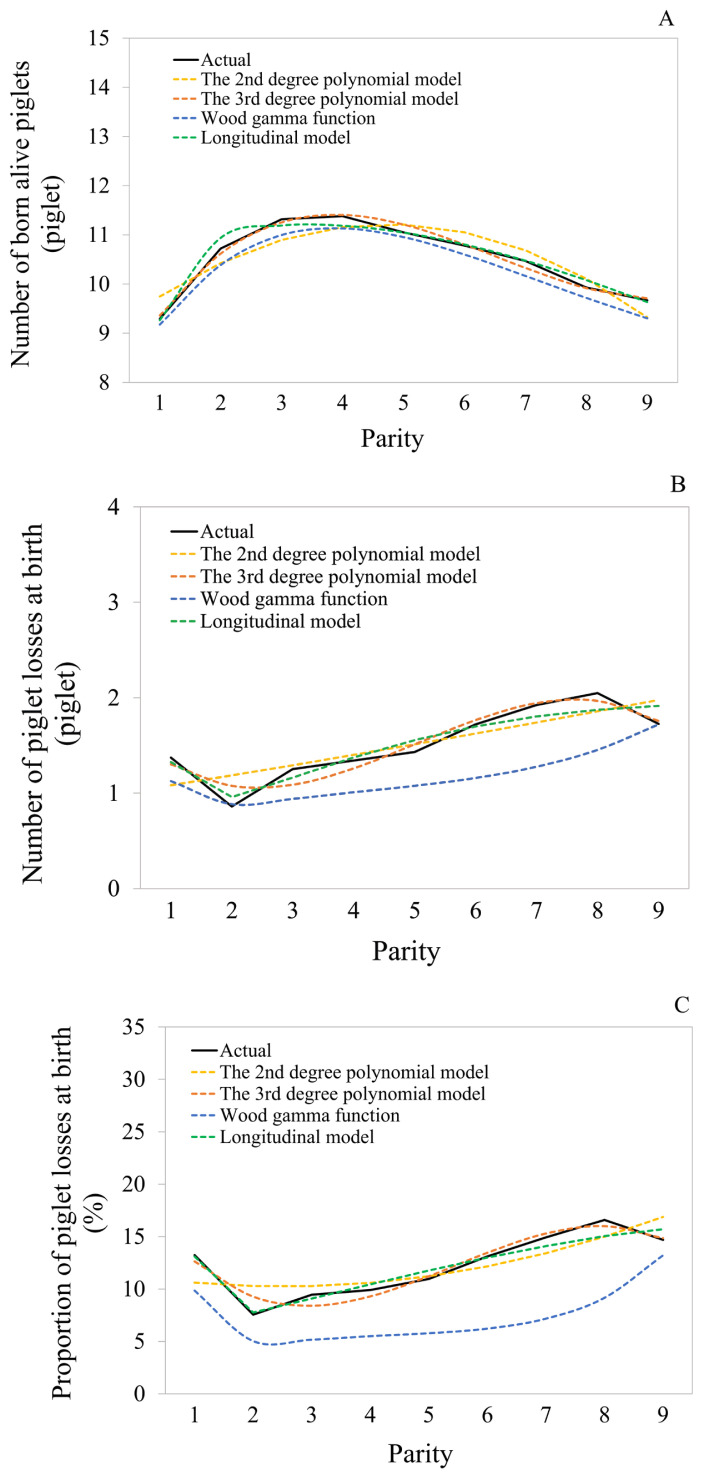
Curves of mean for the actual and the predicted values of individual sows from the 1st to the 9th parities: (A) number of born alive piglets, (B) number of piglet losses at birth, and (C) proportion of piglet losses at birth.

The mean values for actual NBA (Figure 2A), NPL (Figure 2B), and PPL (Figure 2C) across parities were plotted alongside mean values for predicted outcomes generated by four distinct models. These predicted lines illustrated the variations in prediction accuracy across different parities. After assessing the mean and SD of the differences between predicted and actual values across all parities (Table 2), the 3rd degree polynomial model was identified as the optimal model for describing the evolving patterns of NBA, NPL, and PPL from the 1st to the 9th parity due to its ability to achieve the lowest mean and SD in those differences. It's worth noting that there were no significant differences in the use of the 2nd and 3rd degree polynomial models and the longitudinal model for predicting NBA, NPL, and PPL (p>0.05). However, the 3rd degree polynomial model exhibited the lowest SD (1.96 piglets for NBA, 1.33 piglets for NPL, and 10.22% for PPL) for the differences between predicted and actual values across all parities, signifying a higher level of prediction accuracy for all traits. Conversely, utilizing the Wood gamma function for prediction resulted in underestimations for all traits (−0.24±2.26 piglets for NBA, −0.34±1.52 piglets for NPL, and −4.81%±12.58% for PPL).

To verify the goodness-of-fit at population level among the three models exhibiting the lowest mean of the differences between predicted and actual values, AIC and BIC values were employed as criteria for model selection. The goodness-of-fit statistics presented in Table 3 reveal that the 3rd degree polynomial model had the smallest AIC and BIC values for all traits, suggesting it as the most suitable model for describing the patterns of the trait across parities in this Landrace population. Based on the estimated average parameters (***b****_0_*, ***b****_1_*, ***b****_2_*, and ***b****_3_*) derived from the 3rd degree polynomial model, predictive equations for NBA, NPL, and PPL based on sow’s parity (***t***) were established as follows:


$$\begin{array}{l} \text{Predicted NBA}=7.35+2.44\text{t}-0.45{\text{t}}^{2}+0.02{\text{t}}^{3}\\ \text{Predicted NPL}=1.84-0.73\text{t}+0.20{\text{t}}^{2}-0.01{\text{t}}^{3}\\ \text{Predicted PPL}=19.18-8.40\text{t}+1.96{\text{t}}^{2}-0.12{\text{t}}^{3} \end{array}$$

However, it is important to note that the prediction accuracy of the 3rd degree polynomial model displayed variations across different parities, as summarized in Table 4. The means of the differences between the predicted and actual values ranged from −0.14 (7th parity) to 0.16 (5th parity) piglets for NBA, −0.16 (3rd parity) to 0.22 (2nd parity) piglets for NPL, and −1.05 (3rd parity) to 1.72% (2nd parity) for PPL.

## DISCUSSION

The investigation revealed intriguing patterns in piglet loss at birth (NPL and PPL) and litter size (NBA) across different parities among Landrace sows in the study. While piglet loss at birth was notably higher in 1st parity sows, it did not exhibit significant differences compared to later parities, except for NPL in the 8th parity (Table 1). This observation can be attributed to the relatively high SD of NPL and PPL in 1st parity sows, suggesting differences in maturity and readiness for piglet production within this specific subgroup. However, as sows progressed to later parities, the variations in both NPL and PPL tended to decrease. Notably, between the 2nd and 5th parities, the range of PPL was narrower, ranging from 77% to 90%, compared to the 1st and high parities (between the 6th and 9th parities), where the range extended to 100%. This indicates that sows between the 2nd and 5th parities exhibited more consistent sexual maturity and enhanced piglet productivity. Conversely, high parity sows displayed elevated NPL and PPL with increased variation, especially in the 8th parity. This suggests that fertility deterioration in individual sows may manifest at varying levels and times, resulting in greater variation in piglet loss at birth among high parity sows.

A similar trend of high variation was also observed in NBA for 1st and high parity sows, likely for similar reasons as piglet loss at birth. The low piglet productivity and unpredictable performance in 1st and high parity sows underscore the importance of ensuring puberty and readiness in replacement gilts, along with the implementation of culling plans for sows with subpar reproductive performance at high parities. Ensuring uniform and predictable piglet productivity would significantly benefit farm management and piglet production planning, including herd structure management, ultimately leading to enhanced piglet production and overall profitability.

In terms of NBA, NPL, and PPL patterns, the period spanning from the 2nd to the 6th parities showcased the highest piglet production, primarily due to increased NBA. From an efficiency perspective, sows at the 2nd to 4th parities exhibited optimal piglet productivity due to low piglet loss at birth. Despite the noticeable increase in NPL and PPL since the 5th parity, there was no significant difference in NBA between the 2nd and 6th parities. These patterns suggest that Landrace sows, especially under tropical conditions, should ideally be retained in the breeding herd until they have produced at least 6 litters. However, the litter size in later parities did not decline to an extent that warranted culling. As long as the sows remain healthy and reproductively sound without experiencing reproductive failure, which is a common reason for sow removal in tropical areas [20,21], they should be retained in the breeding herd.

Nevertheless, the poor reproductive performance observed in 1st parity sows in this study raises concerns regarding herd structure management. Previous research by Jirattikanpan et al [22] reported a negative correlation between NBA and the proportion of 1st parity sows (r = −0.64; p<0.05), suggesting that a high proportion of 1st parity sows could negatively impact overall piglet production. Effective nutritional management aimed at improving puberty and ensuring adequate body energy reserves for replacement gilts could enhance their performance during the 1st parity [23]. Similarly, the significant increase in PPL observed in the 5th parity is noteworthy, as it indicates suboptimal efficiency in piglet production. Therefore, increased attention and care during gestation and farrowing should be provided to the sows, especially after their 5th litter, to reduce piglet loss at birth, particularly due to mummification and stillbirth.

The observed patterns of NBA, NPL, and PPL in this study can be attributed to the physiological mechanisms of the sows, including ovulation rate, oocyte fertilization rate, and embryo survival and development, which are known to vary with age and parity [24]. Notably, 1st parity sows tend to have lower ovulation rates compared to multiparous sows [25], potentially leading to limitations in litter size due to the immaturity of the uterus. This results in inadequate space for the embryos and affects fetal development and survival [9]. Furthermore, the smaller birth canal size in 1st parity sows could contribute to a higher incidence of stillborn piglets [26]. Additionally, gestating gilts, producing their first litter, may be more susceptible to environmental stressors than mature sows, which can lead to increased embryonic and fetal mortality [27]. These physiological factors collectively contribute to smaller litter size and higher piglet loss in 1st parity sows.

In sows beyond their 1st parity, particularly those in their 6th parity or later, the decrease in NBA and increase in NPL and PPL may be attributed to uterine aging, resulting in reduced muscular tone and efficiency during the farrowing process [10]. Furthermore, high ovulation rates could result in uterine crowding, which can adversely affect placental development, embryo and fetal survival, and placental efficiency [24]. Placental efficiency is closely related to placental-fetal blood flow and nutrient supply from the mother to the fetuses. Crowded uterine conditions may compromise placental development, leading to incomplete fetal development and gestational mortality [28,29]. Research by Borges et al [30] demonstrated that embryos in sows with low placental efficiency were at a higher risk of mummification compared to those in sows with high placental efficiency.

In summary, the observed litter size patterns in this Landrace population, with the highest NBA occurring at the 4th parity, align with findings from various studies conducted in different geographic locations and with different sow breeds [2,10,31]. While the specific parity at which the peak NBA is observed may vary among populations [32,33], the consistent trend of reduced litter size in 1st parity sows is a recurring observation [10,31–34]. Similarly, patterns of NPL and PPL exhibited variations among populations [1,10,32,33], particularly in the 1st parity, likely due to differences in genetic backgrounds, management practices, and environmental conditions, all of which can influence the puberty and fertility of replacement gilts.

The significant variations in NBA, NPL, and PPL observed among sow parities underscore a relatively weak association between reproductive performance and parity. These variations are consistent with low repeatability estimates reported in previous research conducted in the same population [14] and in other commercial populations [11–13]. These low repeatability estimates, along with the low positive genetic correlation between NBA at 1st parity and subsequent litters [35], highlight the limited predictability of reproductive performance based solely on a sow’s initial performance. As a result, it emphasizes the necessity of continuous recording of reproductive traits throughout a sow’s lifetime production as a more reliable criterion for selection.

The occurrence of high piglet loss at birth, both in terms of number (NPL) and proportion (PPL), resulting in small litter size in 1st parity and reduced piglet productivity in high parities (7th parity and beyond), underscores the need for special attention during these periods to enhance sow performance. These observed patterns can be instrumental in guiding farm management decisions aimed at optimizing sow performance and maintaining desired levels of piglet production. Moreover, such management decisions can potentially mitigate economic losses associated with maintaining older sows exhibiting subpar reproductive performance in the breeding herd. By considering the patterns of NBA, NPL, and PPL, pig producers can effectively manage their herd structure, including setting the proportion of sows in each parity in the breeding herd. Given the low piglet production in 1st parity and high parity sows, these sows should be maintained in a lower proportion, while a higher proportion should be allocated to sows in the 2nd to 6th parities, which typically represent the most productive period in their reproductive lifespan. However, it is important to note that patterns of litter size and piglet loss at birth may evolve over time due to various factors such as selection response, farm management practices, and environmental conditions. Therefore, continuous collection of sow records and frequent data re-analysis are warranted to effectively monitor farm productivity and develop suitable replacement plans based on the most current situation.

### Model for predicting litter size and piglet loss at birth

This study, conducted on a Landrace population, identified the 3rd degree polynomial model as the most suitable model for describing litter size and piglet loss at birth during the sow's lifetime production period. However, it is crucial to acknowledge that any model or mathematical function used for predicting sow reproductive performance can yield both over- and under-estimations. Minimizing prediction errors is essential, and this can be achieved by selecting the model with the smallest residual, determined by comparing the difference between predicted and actual values of the trait. In this context, the 3rd degree polynomial model emerged as the optimal choice (Table 2). These findings align with a study by Sell-Kubiak et al [9], which also employed the 3rd degree polynomial model (with Legendre polynomials tested ranging from 1st to 7th order) to fit the parity curve of litter size in Large White sows for genetic parameter estimation. Additionally, the 3rd degree polynomial model was utilized to describe the relationships between parity and the numbers of total born piglets, born alive piglets, and born dead piglets in a study by Ju et al [10]. The simplicity and practicality of the polynomial model make it a valuable tool for pig producers to predict litter size and piglet loss at birth across different parities.

The 3rd degree polynomial model demonstrated superior predictive performance in high parities. Conversely, the lowest predictive ability of the 3rd degree polynomial model was observed in the 5th parity for NBA, and the 2nd parity for NPL and PPL. Notably, the predictive ability appeared to decline at the parity where the curve reached a turning point. For instance, the highest mean difference between predicted and actual NBA values was observed in the 5th parity, coinciding with a change in the curve's direction (dropout) after reaching its peak. Similarly, for NPL and PPL, the highest mean difference was observed in the 2nd parity, followed by the 3rd parity, where the curve dropped to its lowest point in the 2nd parity and then rose again in the 3rd parity. The reduced accuracy of prediction at the turning point of the curve could potentially be attributed to the unique patterns exhibited by each sow. The turning point of the curve may vary depending on the sow's genetic makeup and response to the environment. Genetic and environmental factors could result in distinct physiological changes in sows during the early (maturation) and late (deterioration) stages of their herd life, making accurate predictions of these turning points challenging.

Therefore, caution should be exercised when applying the 3rd degree polynomial model to predict litter size in the 5th parity and piglet loss at birth in the 2nd parity. Overestimation of NBA, NPL, and PPL in these parities could lead to errors in expected piglet numbers, potentially impacting production planning and herd management negatively. Additionally, the limited sample size in this study might have contributed to the reduced predictive ability at the turning point. Further investigation with a larger dataset may help to minimize prediction errors. Consequently, a greater number of reproductive records from sows are needed to construct the most suitable model and enhance the accuracy of prediction.

The longitudinal model emerges as a viable alternative for predicting NBA, NPL, and PPL, given its high accuracy at the 1st parity and the extreme points (peak and bottom) of the curve. During the early period (1st to 4th parities), the predicted values of NBA, NPL, and PPL obtained from the longitudinal model closely aligned with the actual values, surpassing the accuracy of the 3rd degree polynomial model. However, in high parities, the 3rd degree polynomial model outperformed the longitudinal model in terms of accuracy. The remarkable predictive ability in the 1st parity of the complex model was indicative of the efficacy of the first part (**−*b****_1_**** exp*[−(*t*_2_−1)*b*_2_]**) of the longitudinal model. Refining this model through simple linear regression may enhance its predictive performance in high parity. Nevertheless, practical applicability is paramount, and the simplicity of the model is a crucial consideration. Hence, the 3rd degree polynomial model remains preferable for predicting litter size and piglet loss at birth, as it is not only easy to implement but also does not rely on assumptions about the shape of the parity curve.

The Wood gamma function exhibited a substantial underestimate of predicted values, indicating limited predictive power for capturing the patterns of litter size and piglet loss at birth in the Landrace population. The back-transformation step, involving the use of an exponential function, may have interfered with the prediction ability of individual sows. When the predicted and actual values were analyzed based on log-transformed NPL (or PPL), the mean difference between predicted and actual values was close to zero (0.00± 0.25 piglets for tNBA, 0.00±0.52 piglets for tNPL, and 0.00% ±0.18% for tPPL), indicating the predictive power of the Wood gamma function. However, the use of log-transformed values may not be practical in real-world piglet production planning, as decision-making requires realistic values. Therefore, employing the Wood gamma function in a log-linear form may not be an efficient solution for predicting data that contains numerous zero values, such as piglet loss traits.

In summary, the 3rd degree polynomial model serves as a simple and practical tool for users to determine the patterns of NBA, NPL, and PPL at both individual and population levels. This model holds significant potential for predicting sow performance indicators such as NBA, NPL, and PPL, even in sows that have not yet completed their reproductive lifespan, thereby enabling early selection for replacement. Notably, the utilization of random regression models for genetic evaluation of repeated records on individuals over time, as demonstrated in dairy cattle studies, has shown superior accuracy in estimating breeding values compared to other statistical approaches [36,37]. Therefore, considering the application of a random regression model for genetic evaluation of litter size and piglet loss at birth, the utilization of a 3rd degree polynomial regression could be a viable choice for achieving high accuracy in genetic prediction. However, it is important to note that the choice of model and parameters should be specific to the population under consideration, necessitating fitting of models and estimation of parameters tailored to the utilized population. These findings could serve as a valuable case study for the Landrace population raised under tropical conditions. Nevertheless, further investigation in larger populations and across various breeds is warranted to better understand the patterns of piglet loss among different parities.

## CONCLUSION

In summary, this study on Landrace sows in tropical conditions reveals that the 3rd degree polynomial model is a practical choice for predicting litter size and piglet loss at birth across different parities. It emphasizes the need for strategic herd management, with a focus on the proportion of sows retained in the breeding herd. While early parities exhibited suboptimal performance, mid-parity sows were the most productive. These findings provide valuable insights for farm management and call for ongoing data collection and analysis. Future research with larger populations and diverse breeds will help expand our understanding of piglet loss patterns. Ultimately, this study lays a foundation for improving sow performance, enhancing piglet production, and boosting profitability in swine production systems.

## Figures and Tables

**Table 1:** The descriptive statistics for the number of born alive piglets (NBA), number of piglet losses at birth (NPL), and proportion of piglet losses at birth (PPL) of Landrace sows analyzed by parity

Parity	n	NBA (piglet)			NPL (piglet)			PPL (%)		
		
		Mean	SD	Range	Mean	SD	Range	Mean	SD	Range
1	258	9.29^cd^	3.49	0–15	1.37^abc^	1.95	0–10	13.25^abc^	20.30	0–100
2	258	10.72^ab^	3.04	1–20	0.86^a^	1.29	0–9	7.56^a^	11.90	0–90
3	258	11.32^a^	2.70	2–20	1.25^ab^	1.61	0–10	9.46^ab^	11.65	0–77
4	258	11.38^a^	2.88	2–18	1.34^ab^	1.90	0–13	9.90^ab^	13.58	0–80
5	258	11.05^ab^	2.89	1–18	1.43^bcd^	1.72	0–13	10.96^bc^	12.45	0–87
6	258	10.78^ab^	2.78	0–16	1.72^bcd^	1.89	0–10	13.11^bc^	14.38	0–100
7	258	10.47^bc^	2.92	0–18	1.92^cd^	2.26	0–14	14.92^bc^	16.89	0–100
8	258	9.93^cd^	3.13	0–17	2.05^d^	2.32	0–14	16.59^c^	18.54	0–100
9	258	9.67^d^	3.11	0–21	1.72^bcd^	2.12	0–11	14.69^bc^	18.24	0–100
Total	2,322	10.51	3.08	0–21	1.52	1.95	0–14	12.27	15.85	0–100

SD, standard deviation.

a–d Different superscripts within the column denote statistically significant differences based on a t-test with Bonferroni adjustment at a significance level of p<0.05.

**Table 2:** Difference between predicted and actual values for number of born alive piglets (NBA), number of piglet losses at birth (NPL), and proportion of piglet losses at birth (PPL) of individual sows across all parities

Model^1)^	NBA (piglet)			NPL (piglet)			PPL (%)		
		
	Mean	SD	Range	Mean	SD	Range	Mean	SD	Range
1	0.00^a^	2.19	−7.22 to 10.25	0.00^a^	1.48	−10.20 to 5.00	0.00^a^	11.56	−75.42 to 46.79
2	0.00^a^	1.96	−6.08 to 8.47	0.00^a^	1.33	−8.84 to 5.67	0.00^a^	10.22	−62.90 to 41.80
3	−0.24^b^	2.26	−9.96 to 8.24	−0.34^b^	1.52	−12.62 to 4.31	−4.81^b^	12.58	−97.07 to 45.31
4	0.00^a^	2.02	−6.66 to 11.65	0.00^a^	1.38	−8.91 to 5.76	0.00^a^	10.82	−81.13 to 43.31

SD, standard deviation.

1) Model 1, 2nd degree polynomial model; Model 2, 3rd degree polynomial model; Model 3, Wood gamma function; Model 4, longitudinal model.

a,b Different superscripts within the column denote statistically significant differences based on a t-test with Bonferroni adjustment at a significance level of p<0.05.

**Table 3:** Goodness-of-fit comparison with Akaike information criterion (AIC) and Schwarz Bayesian information criterion (BIC) at the population level for number of born alive piglets (NBA), number of piglet losses at birth (NPL), and proportion of piglet losses at birth (PPL)

Model	NBA		NPL		PPL	
		
	AIC	BIC	AIC	BIC	AIC	BIC
2nd degree polynomial	5,127	5,129	3,049	3,051	12,791	12,793
3rd degree polynomial	5,107	5,110	3,034	3,036	12,771	12,773
Longitudinal	11,698	11,727	9,633	9,661	19,364	19,392

AIC, Akaike information criterion; BIC, Bayesian information criterion.

**Table 4:** Difference between predicted and actual values for number of born alive piglets (NBA), number of piglet losses at birth (NPL), and proportion of piglet losses at birth (PPL) in each parity using the 3rd degree polynomial model for prediction

Parity	NBA (piglet)			NPL (piglet)			PPL (%)		
		
	Mean	SD	Range	Mean	SD	Range	Mean	SD	Range
1	0.07	1.08	−3.18 to 2.76	−0.07	0.60	−2.60 to 2.26	−0.62	5.37	−21.13 to 22.91
2	−0.10	2.42	−5.84 to 8.11	0.22	1.21	−5.24 to 4.96	1.72	11.17	−52.70 to 41.80
3	−0.06	2.09	−6.08 to 6.15	−0.16	1.17	−6.04 to 4.48	−1.05	8.67	−30.27 to 26.60
4	0.03	2.19	−5.45 to 7.83	−0.08	1.40	−8.84 to 2.96	−0.61	10.00	−49.57 to 25.41
5	0.16	2.05	−4.49 to 6.20	0.08	1.41	−6.66 to 3.82	0.26	10.01	−31.23 to 24.55
6	0.02	2.12	−5.98 to 8.47	0.04	1.60	−5.88 to 5.67	0.35	12.13	−62.90 to 41.33
7	−0.14	1.90	−5.92 to 6.19	0.02	1.68	−7.63 to 4.76	0.37	12.47	−52.88 to 40.98
8	−0.01	2.22	−5.48 to 7.83	−0.08	1.66	−8.44 to 4.53	−0.59	13.09	−56.62 to 38.12
9	0.04	1.02	−2.88 to 2.90	0.03	0.72	−1.80 to 4.06	0.17	5.84	−20.60 to 24.13

SD, standard deviation.

## Data Availability

The data used in this study will be shared upon a reasonable request to the corresponding author.

## References

[1] Bono C, Cornou C, Lundbye-Christensen S, Kristensen AR (2014). Dynamic production monitoring in pig herds III: modeling and monitoring mortality rate at herd level. Livest Sci.

[2] Hagan JK, Etim NN (2019). The effects of breed, season and parity on the reproductive performance of pigs reared under hot and humid environments. Trop Anim Health Prod.

[3] Piñán J, Alegre B, Kirkwood RN (2021). Effect of season and parity on reproduction performance of Iberian sows bred with Duroc semen. Animals (Basel).

[4] Zhang T, Wang LG, Shi HB (2016). Heritabilities and genetic and phenotypic correlations of litter uniformity and litter size in Large White sows. J Integr Agric.

[5] Jaichansukkit T, Suwanasopee T, Koonawootrittriron S, Tummaruk P, Elzo MA (2017). Effect of daily fluctuations in ambient temperature on reproductive failure traits of Landrace and Yorkshire sows under Thai tropical environmental conditions. Trop Anim Health Prod.

[6] Klimas R, Klimienė A, Sobotka W, Kozera W, Matusevičius P (2020). Effect of parity on reproductive performance sows of different breeds. S Afr J Anim Sci.

[7] Lavery A, Lawlor PG, Magowan E, Miller HM, O’Driscoll1 K, Berry DP (2019). An association analysis of sow parity, live-weight and back-fat depth as indicators of sow productivity. Animal (Basel).

[8] Nevrkla P, Lujka J, Kopec T (2021). Combined effect of sow parity and terminal boar on losses of piglets and pre-weaning growth intensity of piglets. Animals (Basel).

[9] Sell-Kubiak E, Knol EF, Mulder HA (2019). Selecting for changes in average “parity curve” pattern of litter size in Large White pigs. J Anim Breed Genet.

[10] Ju M, Wang X, Li X (2022). Effects of litter size and parity on farrowing duration of Landrace × Yorkshire sows. Animals (Basel).

[11] Jaichansukkit T, Suwanasopee T, Koonawootrittriron S (2014). Genetic parameters for litter traits at birth of Landrace sow populations raised under Thai tropical conditions. Thai J Anim Sci.

[12] Ye J, Tan C, Hu X, Wang A, Wu Z (2018). Genetic parameters for reproductive traits at different parities in Large White pigs. J Anim Sci.

[13] Ogawa S, Konta A, Kimata M, Ishii K, Uemoto Y, Satoh M (2019). Estimation of genetic parameters for farrowing traits in purebred Landrace and Large White pigs. Anim Sci J.

[14] Thiengpimol P, Koonawootrittriron S, Suwanasopee T (2020). Genetic parameters for proportion of piglet loss at birth in a Landrace population. Agric Nat Resour.

[15] Toft N, Jørgensen E (2002). Estimation of farm specific parameters in a longitudinal model for litter size with variance components and random dropout. Livest Prod Sci.

[16] Statistical Analysis System (SAS) (2011). SAS® 93 system options.

[17] Wood PDP (1967). Algebraic model of the lactation curve in cattle. Nature.

[18] Akaike H (1974). A new look at the statistical model identification. IEEE Trans Automat Contr.

[19] Schwarz G (1978). Estimating the dimension of a model. Ann Stat.

[20] Segura-Correa JC, Ek-Mex E, Alzina-López A, Segura-Correa VM (2011). Frequency of removal reasons of sows in Southeastern Mexico. Trop Anim Health Prod.

[21] Masaka L, Sungirai M, Nyamukanza C, Bhondai C (2014). Sow removal in a commercial pig herd in Zimbabwe. Trop Anim Health Prod.

[22] Jirattikanpan N, Suwanasopee T, Koonawootrittriron S, Elzo MA (2016). Association between parity proportion and piglet production in Landrace sows. Kaen Kaset.

[23] Kyriazakis I, Whittemore CT (2006). Whittemore’s science and practice of pig production.

[24] Kemp B, Da Silva CLA, Soede NM (2018). Recent advances in pig reproduction: focus on impact of genetic selection for female fertility. Reprod Dom Anim.

[25] Belstra BA (2003). Parity associated changes in reproductive performance: physiological basis? [Internet].

[26] Cowart RP, Youngquist RS, Threlfall WR (2007). Parturition and dystocia in swine. Current therapy in large animal theriogenology.

[27] Bloemhof S, Mathur PK, Knol EF, van der Waaij EH (2013). Effect of daily environmental temperature on farrowing rate and total born in dam line sows. J Anim Sci.

[28] Campos PHRF, Silva BAN, Donzele JL, Oliveira RFM, Knol EF (2012). Effects of sow nutrition during gestation on within-litter birth weight variation: a review. Animal (Basel).

[29] Pardo CE, Bérard J, Kreuzer M, Bee G (2013). Intrauterine crowding impairs formation and growth of secondary myofibers in pigs. Animal (Basel).

[30] Borges VF, Bernardi ML, Bortolozzo FP, Wentz I (2005). Risk factors for stillbirth and foetal mummification in four Brazilian swine herds. Prev Vet Med.

[31] Sasaki Y, McTaggart I, Koketsu Y (2011). Assessment of lifetime economic returns of sows by parity of culled sows in commercial breeding herds. J Vet Epidemiol.

[32] Tantasuparuk W, Lundeheim N, Dahn AM, Kunavongkrit A, Einarsson S (2000). Reproductive performance of purebred Landrace and Yorkshire sows in Thailand with special reference to seasonal influence and parity number. Theriogenology.

[33] Vargovic L, Harper JA, Bunter KL (2022). Traits defining sow lifetime maternal performance. Animals (Basel).

[34] Plaengkaeo S, Duangjinda M, Stalder KJ (2021). Identifying early indicator traits for sow longevity using a linear-threshold model in Thai Large White and Landrace females. Anim Biosci.

[35] Noppibool U, Elzo MA, Koonawootrittriron S, Suwanasopee T (2017). Genetic correlations between first parity and accumulated second to last parity reproduction traits as selection aids to improve sow lifetime productivity. Asian-Australas J Anim Sci.

[36] Schaeffer LR (2004). Application of random regression models in animal breeding. Livest Prod Sci.

[37] Oliveira HR, Brito LF, Lourenco DAL (2019). Invited review: advances and applications of random regression models: from quantitative genetics to genomics. J Dairy Sci.

